# Mental Health Themed Student Selected Components: A Strategy To Increase Recruitment Into Psychiatric Training

**DOI:** 10.1192/j.eurpsy.2025.2381

**Published:** 2025-08-26

**Authors:** T. I. D. Parry, D. Levy, S. De Souza

**Affiliations:** 1 Medical Education, Somerset Foundation Trust, Taunton; 2 University of Bristol, Bristol; 3 Somerset Foundation trust, Taunton, United Kingdom

## Abstract

**Introduction:**

The University of Bristol Medical School, United Kingdom, has student-selected components (SSC) making up a substantial proportion of its curriculum. This practice is common among UK medical schools. SSCs can inspire interest in specialities that students may have less exposure to during undergraduate training, such as psychiatry. Psychiatry has a broad range of sub-specialities and themes which can be explored in an SSC. The author supervised one such project in which a student researched and produced a series of podcasts about the science of happiness.

**Objectives:**

To explore the use of student-selected components (SSC) in increasing exposure to psychiatry in the undergraduate curriculum

To explore the impact of student-selected components (SSC) in increasing recruitment into psychiatric training

**Methods:**

An initial literature review was performed with the following keywords using Medline on OvidSP.

**Results:**

Fourteen papers addressed the use of psychiatry student-selected components (SSC) in undergraduate medical education and their influence on career specialty choice.

**Conclusions:**

Student-selected components (SSC) are an important strategy for increasing exposure to psychiatry in undergraduate medical education and recruitment into psychiatry.

**Keywords:**

Medical undergraduate education, elective, student-selected components, special study modules, psychiatry, mental health.

**Disclosure of Interest:**

None Declared

